# Myocardial strain assessment in the human fetus by cardiac MRI using Doppler ultrasound gating and feature tracking

**DOI:** 10.1007/s00330-023-10551-0

**Published:** 2024-01-10

**Authors:** Maryam Dargahpour Barough, Manuela Tavares de Sousa, Bettina Hergert, Roland Fischer, Lukas Huber, Jan Moritz Seliger, Michael Gerhard Kaul, Gerhard Adam, Jochen Herrmann, Peter Bannas, Bjoern P. Schoennagel

**Affiliations:** 1 Department of Diagnostic and Interventional Radiology and Nuclear Medicine, University Medical Center Hamburg-Eppendorf, Martinistrasse 52, 20251 Hamburg, Germany; 2 Department of Obstetrics and Fetal Medicine, University Medical Center Hamburg-Eppendorf, Martinistrasse 52, 20251 Hamburg, Germany; 3 Department of Diagnostic and Interventional Radiology and Nuclear Medicine, Section of Pediatric Radiology, University Medical Center Hamburg-Eppendorf, Martinistrasse 52, 20251 Hamburg, Germany

**Keywords:** Fetal heart, Cine magnetic resonance imaging, Cardiac-gated imaging techniques, Global longitudinal strain

## Abstract

**Objectives:**

Assessment of myocardial strain by feature tracking magnetic resonance imaging (FT-MRI) in human fetuses with and without congenital heart disease (CHD) using cardiac Doppler ultrasound (DUS) gating.

**Methods:**

A total of 43 human fetuses (gestational age 28–41 weeks) underwent dynamic cardiac MRI at 3 T. Cine balanced steady-state free-precession imaging was performed using fetal cardiac DUS gating. FT-MRI was analyzed using dedicated post-processing software. Endo- and epicardial contours were manually delineated from fetal cardiac 4-chamber views, followed by automated propagation to calculate global longitudinal strain (GLS) of the left (LV) and right ventricle (RV), LV radial strain, and LV strain rate.

**Results:**

Strain assessment was successful in 38/43 fetuses (88%); 23 of them had postnatally confirmed diagnosis of CHD (e.g., coarctation, transposition of great arteries) and 15 were heart healthy. Five fetuses were excluded due to reduced image quality. In fetuses with CHD compared to healthy controls, median LV GLS (− 13.2% vs. − 18.9%; *p* < 0.007), RV GLS (− 7.9% vs. − 16.2%; *p* < 0.006), and LV strain rate (1.4 s^−1^ vs. 1.6 s^−1^; *p* < 0.003) were significantly higher (i.e., less negative). LV radial strain was without a statistically significant difference (20.7% vs. 22.6%; *p* = 0.1). Bivariate discriminant analysis for LV GLS and RV GLS revealed a sensitivity of 67% and specificity of 93% to differentiate between fetuses with CHD and healthy fetuses.

**Conclusion:**

Myocardial strain was successfully assessed in the human fetus, performing dynamic fetal cardiac MRI with DUS gating. Our study indicates that strain parameters may allow for differentiation between fetuses with and without CHD.

**Clinical relevance statement:**

Myocardial strain analysis by cardiac MRI with Doppler ultrasound gating and feature tracking may provide a new diagnostic approach for evaluation of fetal cardiac function in congenital heart disease.

**Key Points:**

*• MRI myocardial strain analysis has not been performed in human fetuses so far.*

*• Myocardial strain was assessed in human fetuses using cardiac MRI with Doppler ultrasound gating.*

*• MRI myocardial strain may provide a new diagnostic approach to evaluate fetal cardiac function.*

## Introduction

Myocardial strain is a sensitive prognostic marker of systolic dysfunction in children and adults [1–3]. In the fetus, however, strain analysis using magnetic resonance imaging (MRI) was not possible due to technical limitations. Only recently, dynamic fetal cardiovascular MRI using an external MR-compatible Doppler ultrasound (DUS) device for fetal cardiac gating has been successfully applied. The DUS device allowed anatomical and functional imaging of the fetal cardiovascular system with high spatio-temporal resolution [4, 5]. Therefore, we aimed to perform dynamic fetal cardiac MRI using DUS gating for assessment of myocardial strain analysis using feature tracking (FT-) MRI.

Application of fetal cardiovascular MRI was formerly limited by the lack of a cardiac gating signal, which is necessary to allow for dynamic imaging with sufficient spatio-temporal resolution. The development of an external MR-compatible DUS sensor for the detection of the fetal heartbeat and the generation of a cardiac gating signal provides a promising solution [6]. Since its introduction few years ago, DUS gating realized innovative applications of cardiovascular MRI in the human fetus, i.e., anatomical and functional analysis [7–10].

FT-MRI is a widespread method for the assessment of myocardial strain and enables analysis of systolic contraction patterns using standard cine MR images [1]. Global longitudinal strain (GLS) allows early detection of myocardial impairment even at subclinical levels, and is predictive for cardiac adverse events in several cardiac diseases [2, 11]. Strain analysis using FT-MRI in the human fetus could add to a better understanding of cardiac pathophysiology in fetuses with suspected congenital heart disease (CHD) and therefore provide clinically relevant information.

The aim of this study was to assess myocardial strain by FT-MRI in human fetuses with and without CHD using cardiac DUS gating.

## Material and methods

### Study population

The local ethics committee approved this fetal cardiovascular MRI study design. Both fetuses with and without echocardiographic suspicion of CHD were included in the study and all fetal cardiovascular MR exams were performed as part of the study. All pregnant women gave written informed consent to participate in the study. Inclusion criteria were willingness of pregnant women to participate in the study and to undergo fetal cardiac MRI, independent of suspected diagnosis of prenatal ultrasound screening. Exclusion criteria were missing consent to participate in the study or general contraindications for MRI (e.g., claustrophobia).

We identified 43 fetal cardiac MRI records (between December 2020 and November 2022) with acquired 4-chamber views. Fetal median gestational age was 34.4 weeks (range 28–41 weeks) and median maternal age was 33.7 years (range 24–41 years).

### Fetal cardiac MRI

MRI was performed on a 3-T MR scanner (Ingenia Elition, Philips Medical Systems) using a 32-channel phased array torso coil placed on the maternal abdomen. Depending on personal preference, pregnant women were placed in supine or lateral position. For fetal cardiac gating, a MR-compatible DUS sensor (smart-sync, Northh Medical GmbH) was positioned on the pregnant woman’s abdomen and fixed with an elastic band. The detected DUS signal of the fetal heart serves as the gating signal that is transferred to the physiological MR [4].

After a survey scan, the orientation of the fetal heart was depicted by a series of three orthogonal planes of a T2-weighted turbo spin echo sequence (FOV 350 × 300 mm^2^, matrix 292 × 216, 15 slices, slice thickness 4 mm, slice gap 0.4 mm, TR 2450 ms, TE 80 ms, flip angle 120°, echo train length 105, SENSE factor 2), each within 36 s acquisition time.

Functional cardiac cine imaging using DUS gating was performed in maternal breath hold technique acquiring a two-dimensional k-space segmented multi-shot balanced steady-state free-precession (bSSFP) sequence in 4-chamber view orientation (FOV 246 × 246 mm^2^, matrix size 164 × 164, 12 slices with thickness 5 mm and gap − 1 mm, TR 3.9 ms, TE 1.96 ms, flip angle 60°, 12 shots with echo train length of 20, and shot duration of 78.6 ms). Similar to adult cardiac MRI, fetal 4-chamber views were planned from acquired pseudo-axis views. Scan time per breath hold was 14 s for cine-bSSFP 4-chamber views. Reconstructed voxel size of cine images was 0.96 × 0.96 × 5 mm^3^.

Average fetal heart rate in our cohort was 136 bpm, with an average RR interval length of 440 ms. With an average of 22 reconstructed phases per cardiac cycle, the temporal resolution of the applied cine-bSSFP sequence was 21.6 ms, corresponding to a frame rate of 46 per second.

### Strain analysis

Myocardial strain was determined on the acquired 4-chamber cine images of the fetal heart using a dedicated feature tracking software (Segment, Version 2.1.R.6108, Medviso). The software determines myocardial strain by computing interframe deformation fields by an endocardial tracking strategy based on non-rigid image registration [12]. The entire image content (i.e., blood pool, entire myocardium) is used during the optimization process instead of tracking only the myocardial boundaries [12]. For this study, the software algorithm was optimized for fetal specifications by the vendor. Global longitudinal strain (GLS) from both the left (LV) and right ventricle (RV), LV strain rate, and LV radial strain were calculated.

**Fig. 1:**
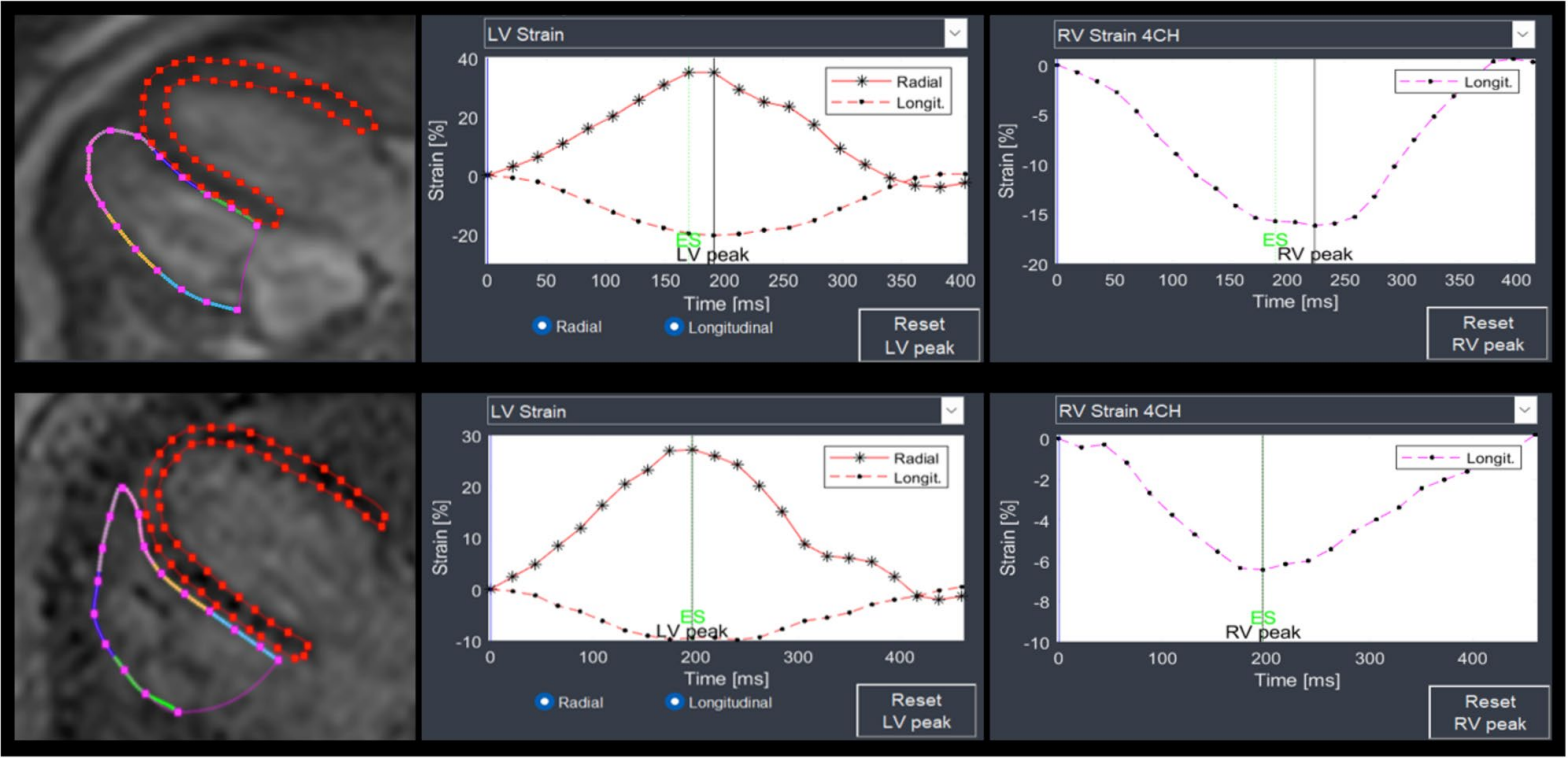
Assessment of strain parameters from fetal cardiac MRI cine 4-chamber views. Upper row: Contouring of endo- and epicardial boundaries of the left ventricle (LV, red) and the right ventricle (RV, colored line) with inclusion of the tricuspid valve plane in fetal cardiac MRI end-diastolic 4-chamber view of a healthy fetus (gestational age, 36^+6^ weeks). The software automatically propagates defined contours to calculate LV and RV strain parameters: LV GLS − 20.5%, RV GLS − 17.1%, radial strain 34.9%, strain rate 1.70 s^−1^. Bottom row: Fetal cardiac MRI end-diastolic 4-chamber view with LV and RV contouring in a fetus with CHD (diagnosis: TGA, gestational age, 36^+2^ weeks). LV and RV GLS and strain rate were increased (i.e., less negative) while radial strain was normal: LV GLS − 9.4%, RV GLS − 6.5%, radial strain 27.2%, strain rate 0.84 s^−1^

The endocardial contour of the RV and the tricuspid valve plane was manually delineated. The LV myocardium was acquired by delineation of the endocardial and epicardial contour. This manual delineation of endo- and epicardial contours on end-diastolic images was followed by an automatic propagation throughout the cardiac cycle, generating myocardial strain and strain rate curves [12]. In the case of suboptimal automatic tracking, endo- and epicardial contours were manually adjusted and re-propagated. Contouring was performed in consensus by two operators with 4 years (M.D.) and 14 years (B.S.) of expertise in cardiac MRI, respectively (Fig. 1).

### Statistical analysis

All results are described as median value and 95% range. Differences between fetuses with CHD and healthy control fetuses were assessed by the Mann–Whitney *U*-test. Correlations of parameters were assessed by Spearman rank correlation coefficients (*r*_S_). Uni- and bivariate discriminant analysis of strain parameters was applied to separate healthy controls from CHD fetuses using a priori equal group size, which adjusts different CHD (*n* = 23) and control numbers (*n* = 15) to the same statistical level (STATISTICA v. 6.1, Stat-Soft Inc.). *p*-values < 0.05 were considered statistically significant.

## Results

Cine 4-chamber views were successfully acquired in 38 of the 43 fetuses (88.4%) and included for further analyses. Five cases (11.6%) were excluded due to fetal movements that resulted in insufficient cardiac gating and poor image quality. In these cases, the feature tracking software was unable to track the contours of the ventricles over the cardiac cycle.

In 23/38 fetuses (60.5%), CHD was confirmed by postnatal echocardiography. Diagnoses of CHD included the following: atrial and/or ventricular septal defects (*n* = 9), valvular anomaly (*n* = 6), aortic arch anomaly/coarctation (*n* = 5), transposition of the great arteries (*n* = 3), univentricular heart (*n* = 2), tetralogy of Fallot (*n* = 1), and Ebstein anomaly (*n* = 1). As combinations of anomalies were observed, their total number is higher than number of fetuses with CHD.

In two cases of a univentricular left ventricle, analysis of the RV was not possible. The remaining 15 fetuses (39.5%) were healthy controls without cardiovascular abnormalities by postnatal echocardiography.

In healthy control fetuses, there was no correlation with gestational age for LV GLS, RV GLS, strain rate, or radial strain (all *p* > 0.12, gestational age range 28–41 weeks). Significant correlations were only observed between LV GLS and strain rate (*r*_S_ = 0.75, *p* = 0.001) as well as radial strain (*r*_S_ =  − 0.54; *p* = 0.038), but not with RV GLS (*p* = 0.2). In control fetuses, LV GLS was significantly lower when compared to RV GLS (*p* = 0.015).

### Comparison of strain parameters in fetuses with CHD and healthy controls

**Fig. 2:**
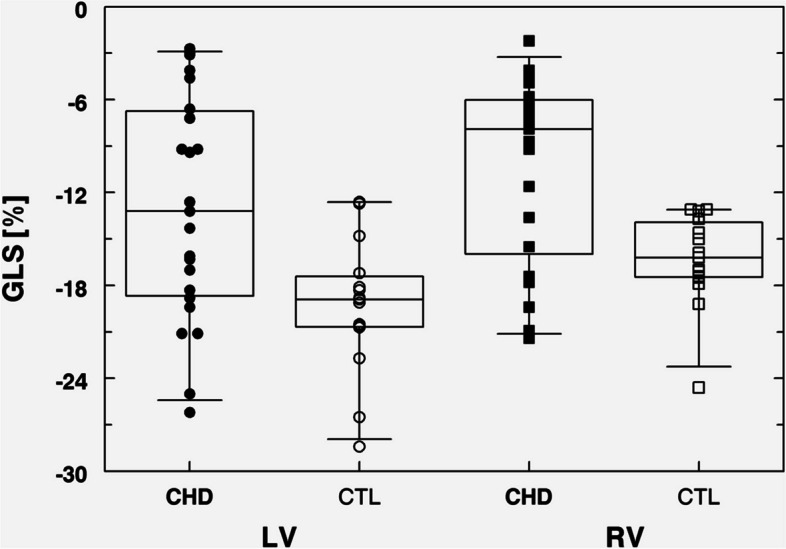
Comparison of global longitudinal strain (GLS) of left (LV) and right ventricle (RV) in fetuses with congenital heart disease (CHD) and healthy controls (CTL). Data are presented as box-whisker plot (median, inter-quartile range, 95% range). Both LV GLS (*p* < 0.007) and RV GLS (*p* < 0.006) are significantly higher in fetuses with CHD than in CTL

Fetuses with CHD had a higher (i.e., less negative) median LV GLS (− 13.2% [range − 3 to − 26%] vs. − 18.9% [range − 13 to − 28%]) (*p* < 0.007) when compared with healthy control fetuses. Median RV GLS was also significantly higher in fetuses with CHD when compared with controls (− 7.9% [range − 3 to − 21%] vs. − 16.2% [range − 13 to − 23%]) (*p* < 0.006) (Fig. 2; Table 1).

**Table 1:** Demographic and systolic strain parameters for fetuses with congenital heart disease (CHD) and healthy control fetuses (CTL)^a^

Parameter	CHD	CTL	*p*
*n*	23	15	na
Gestational age (weeks)	36.3 (33, 38)	35.0 (28, 41)	0.48
Maternal age (years)	31.7 (23, 41)	35.0 (26, 40)	0.12
LV GLS (%)	− 13.2 (− 26, − 3)	− 18.9 (− 28, − 13)	0.007
RV GLS (%)^b^	− 7.9 (− 21, − 3)	− 16.2 (− 23, − 13)	0.006
LV strain rate (s^−1^)	1.4 (2.1, 0.2)	1.6 (2.4, 1.0)	0.002
LV radial strain (%)	20.7 (3, 39)	22.6 (11, 46)	0.11

^a^Data are presented as median within 95% range and significance levels *p* were tested by the Mann–Whitney *U*-test

^b^Number of fetuses with analysis of RV GLS was *n* = 21 as two fetuses had univentricular heart

Median LV strain rate in fetuses with CHD was − 1.4 s^−1^ (range 0.1 to 2.3 s^−1^) and significantly higher when compared to controls with a strain rate of 1.6 s^−1^ (range 1.0 to 2.4 s^−1^) (*p* < 0.003).

There was no significant difference of LV radial strain when comparing fetuses with CHD and controls with median values of 20.7% (range 3–39%) and 22.6% (range 11–46%) (*p* = 0.1) (Table 1).

### Discriminant analysis of strain parameters

**Fig. 3:**
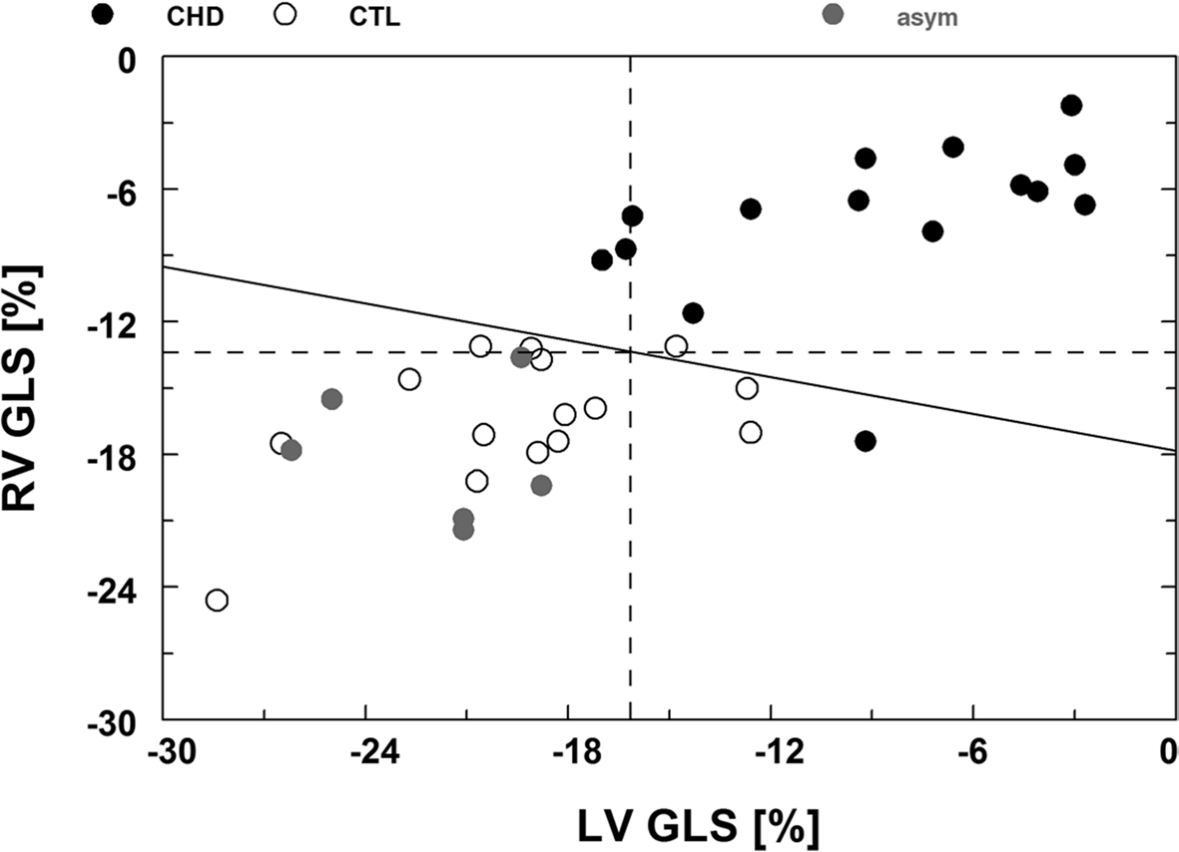
Right ventricular global longitudinal strain (RV GLS) is associated with left ventricular global strain (LV GLS). A linear bivariate discriminant function (solid line) separates healthy control fetuses (CTL: open circles) from fetuses with congenital heart disease (CHD: solid circles) with a sensitivity of 67% and a specificity of 93%. Univariate cut-off thresholds for RV GLS at − 13.4% (dashed horizontal line) and for LV GLS at − 16.2% (dashed vertical line) separate CHD and CTL fetuses with a sensitivity/specificity of 67%/80% and 61%/80%, respectively. Few fetuses with CHD behave like healthy controls (gray circles; asym = non-symptomatic)

Univariate discriminant analysis of LV GLS revealed a sensitivity of 61% and a specificity of 80% to differentiate fetuses with CHD from controls (cut-off threshold at − 16.2%). For RV GLS, a sensitivity of 67% and specificity of 80% were calculated to differentiate between fetuses with CHD and controls (cut-off threshold at − 13.4%). However, applying bivariate discriminant analysis for LV GLS and RV GLS revealed a comparable sensitivity of 67% but a higher specificity of 93% and a cut-off line at LV GLS =  − 17.2 + (− 0.28) ⋅ RV GLS (Fig. 3).

## Discussion

To the best of our knowledge, this is the first study successfully applying dynamic fetal cardiac MRI by DUS gating for assessment of myocardial strain FT-MRI in human fetuses. This functional cardiac MRI study found significant differences in RV and LV strain parameters between fetuses with CHD and healthy controls, suggesting that CHD is associated with prenatal cardiac dysfunction.

Determination of LV and RV GLS was successful in 88% of fetuses in our study. In the remaining fetuses, artifacts in dynamic MR images due to gross fetal motion prevented sufficient myocardial contouring by the software and, therefore, strain analysis was not feasible. This is similar to experiences with speckle tracking echocardiography (STE) where fetal movement with reduced image quality led to comparable success rates between 76 and 98% [13, 14].

The subgroup of 15 control fetuses in our cohort revealed median LV GLS of − 18.9% and RV GLS of − 16.2% and a median LV strain rate of 1.6 s^−1^. So far, there are no other MRI studies available reporting myocardial strain values in fetuses. Our results can be compared to normal values reported for echocardiography published by van Oostrum et al on the basis of 124 healthy fetuses [15]. Referring to similar gestational age groups (28–41 weeks) and consistent with our results, mean LV GLS between − 21.1 and − 18.4%, RV GLS between − 18.8 and − 16.1%, and LV strain rates between 1.78 and 1.45 s^−1^ were observed by von Oostrum et al [15].

However, a recent meta-analysis reported that echocardiography-derived fetal strain parameters vary widely [16]*.* For a similar gestational age group compared with our cohort, LV GLS between − 13.6 and − 24.7%, RV GLS between − 13.4 and − 23.2%, and LV strain rate between 1.1 and 2.6 s^−1^ were observed [16]. Inconsistency is an inherent issue in myocardial strain analysis for both echocardiography and MRI [17]. In fetuses, the considerable variation of echocardiographic strain results was attributed to different acquisition techniques and applied software [17]. In adults, the lack of standardization results in a wide range of proposed normal limits for LV GLS (16–21%) and for RV GLS (19–24%) [1, 18–20]. Possible reasons for this wide range are influencing factors such as age, gender, and heart rate, but also the use of different MRI methods (e.g., myocardial tagging vs. FT-MRI) and software [21, 22].

In our study, FT-MRI strain analysis revealed significantly increased (i.e., less negative values) LV GLS, RV GLS, and LV strain rate in fetuses with CHD when compared to control fetuses. Interestingly, LV GLS and RV GLS did not correlate, but their combination was more precise for the identification of fetuses with CHD (sensitivity 67%, specificity 93%) than each parameter alone. This finding indicates the additive value of considering strain parameters of both, LV and RV, in the evaluation of fetal cardiac function. Using echocardiography, recent studies suggested that myocardial strain reflects the changing physiology in fetuses with CHD [23–25], e.g., altered strain parameters were observed in fetuses with tetralogy of Fallot [26]. In fetuses with transposition of the great arteries, echocardiographic strain measures predicted urgent postnatal intervention [27]. Alsolai et al demonstrated the usefulness of increased LV GLS and LV strain rate for risk stratification of intrapartum fetal compromise [28]. CHD in our fetuses were heterogeneous, including severe CHD. However, our results indicate that FT-MRI reflects altered physiology in fetuses with CHD by increased LV GLS, RV GLS, and LV strain rate. Of note, LV radial strain did not differ between healthy fetuses and fetuses with CHD in our cohort and the available echocardiographic literature does not report on radial strain in the fetus.

In our healthy control fetuses, LV GLS was significantly lower (i.e., more negative values) compared to RV GLS. In agreement, decreased echocardiography-derived LV GLS during weeks 18–41 of gestation was reported in a cohort of 124 fetuses [15]. Instead, other echocardiography studies reported decreased RV GLS, which was ascribed to different ventricular fiber orientation with predominant longitudinal fibers in the RV but multidirectional fibers in the LV [14]. Employing an echocardiographic high frame rate model, it was concluded that previously observed interventricular differences of strain values in healthy fetal hearts may be related to low temporal resolutions and may not reflect physiological differences [13]. High frame rates above 90 frames per second are recommended in echocardiography to avoid underestimation of time-dependent myocardial deformation [17]. The average temporal resolution of our cine-bSSFP sequence was 21.6 ms (fetal heart rate dependent), corresponding to a calculated frame rate of 46 per second, which may have influenced the calculated differences between the LV and RV GLS. The interventricular relation of strain values remains unclear, and further studies are needed to elucidate the relationship between LV GLS and RV GLS in the fetus. However, the proposed pathophysiological and technical considerations may help to provide sufficient ventricular strain data.

In our cohort, LV GLS and RV GLS were not related to gestational age. There are conflicting results for the relationship of GLS and gestational age in echocardiographic studies. Most echocardiographic studies also demonstrated stable GLS (especially LV GLS) during pregnancy [23, 29, 30]. Some authors observed increasing values (less negative values) with advancing gestation [15], while others showed decrease in RV GLS but stable LV GLS with advancing gestation [14, 31, 32]. These variations are explained by incoherent study designs (e.g., cross-sectional instead of longitudinal) or technical inconsistencies (e.g., non-standardized analysis software) [16]. From a physiological perspective, stable GLS during pregnancy could be assumed from the moment when a stable amount of cardiomyocytes is achieved in midterm pregnancy [32, 33]. As GLS is preload- and afterload-dependent, it is argued that changes during the third trimester can be expected due to changing fetal hemodynamics [13, 16, 34].

A limitation of our study is that, due to the retrospective design, we cannot provide direct comparison of strain parameters with echocardiography. An inherent technical limitation is the reduced temporal resolution of MRI in comparison to available echocardiographic studies, with potential underestimation of strain values. In addition, FT-MRI strain analysis was limited to fetal cardiac 4-chamber views.

In conclusion, myocardial strain was successfully assessed in the human fetus performing dynamic fetal cardiac MRI with DUS gating. Our study indicates that strain parameters may allow for differentiation between fetuses with and without CHD. Therefore, FT-MRI myocardial strain analysis using dynamic fetal cardiac MRI with DUS gating may provide a new diagnostic approach for the evaluation of fetal cardiac function in CHD. Future longitudinal studies with larger fetal populations are warranted for the assessment of reference values and the evaluation of the clinical benefit of this promising approach.

## Abbreviations

bSSFP: Balanced steady-state free-precession
CHD: Congenital heart disease
DUS: Doppler ultrasound
FT: Feature tracking
GLS: Global longitudinal strain
LV: Left ventricle
RV: Right ventricle

## Declarations

### Guarantor

The scientific guarantor of this publication is Bjoern P. Schoennagel.

### Statistics and biometry

One of the authors (RF) has significant statistical expertise.

### Study subjects or cohorts overlap

Twelve of 43 study subjects have been previously reported in a former study with a different topic (*Knapp J *et al,* Eur. Rad. 2023; Fetal 4D flow MRI of the great thoracic vessels at 3 Tesla using Doppler-ultrasound gating: a feasibility study*). This different study did not analyze myocardial strain.

### Methodology

•retrospective

•experimental

•performed at one institution
